# Behavior of the Flexural Strength of Hemp/Polypropylene Composites: Evaluation of the Intrinsic Flexural Strength of Untreated Hemp Strands

**DOI:** 10.3390/polym15020371

**Published:** 2023-01-10

**Authors:** María E. Vallejos, Roberto J. Aguado, Ramón Morcillo-Martín, José A. Méndez, Fabiola Vilaseca, Quim Tarrés, Pere Mutjé

**Affiliations:** 1 Instituto de Materiales de Misiones (IMAM), Universidad Nacional de Misiones—Consejo Nacional de Investigaciones Científicas y Técnicas (UNaM–CONICET), Posadas 3300, Argentina; 2 LEPAMAP-PRODIS Research Group, University of Girona, C/ Maria Aurèlia Capmany, 61, 17003 Girona, Spain; 3 Biopren Group (RNM940), Chemical Engineering Department, Faculty of Science, Universidad de Córdoba, 14014 Córdoba, Spain; 4 Advanced Biomaterials and Nanotechnology (BIMATEC Group), University of Girona, C/Maria Aurèlia Capmany, 61, 17003 Girona, Spain

**Keywords:** biocomposites, flexural strength, lignocellulosics, micromechanics, natural fibers, polypropylene

## Abstract

The growing demand for plant fiber-reinforced composites offers new opportunities to compete against glass fiber (GF)-reinforced composites, but their performance must be assessed, revised, and improved as much as possible. This work reports on the production and the flexural strength of composites from polypropylene (PP) and hemp strands (20–50 wt.%), using maleic anhydride-grafted PP (MAPP) as a compatibilizer. A computational assessment of the reaction between cellulose and MAPP suggested the formation of only one ester bond per maleic anhydride unit as the most stable product. We determined the most favorable MAPP dosage to be 0.06 g per gram of fiber. The maximum enhancement in flexural strength that was attained with this proportion of MAPP was 148%, corresponding to the maximum fiber load. The modified rule of mixtures and the assumption of similar coupling factors for tensile and flexural strength allowed us to estimate the intrinsic flexural strength of hemp strands as 953 ± 116 MPa. While falling short of the values for sized GF (2415 MPa), the reinforcement efficiency parameter of the natural fibers (0.209) was found to be higher than that of GF (0.045).

## 1. Introduction

Composites in which the dispersed phase (reinforcement) consists of natural fibers are commonly named biocomposites, regardless of the matrix. They amounted to a market size of USD 25.4 billion in 2021 and their compound annual growth rate (CAGR) has been projected to be as high as 16% [1]. On average, the global composites market is expected to grow at a 7% rate [2]. Interestingly, the CAGR expected for glass fiber (GF) composites until 2026 is lower than that for hemp, flax, jute, and kenaf [3]. That said, as of today and despite the expectations for natural fiber-reinforced materials, GF-reinforced composites still prevail over them in terms of market size, roughly by a 20:1 ratio [2]. However, natural fibers possess several practical and environmental advantages. They are lighter, renewable, and readily usable for composite manufacturing with little or no consumption of energy and chemicals [4]. In contrast, the production of GF requires high energy inputs, entailing a higher carbon footprint [5].

**Figure 1:**
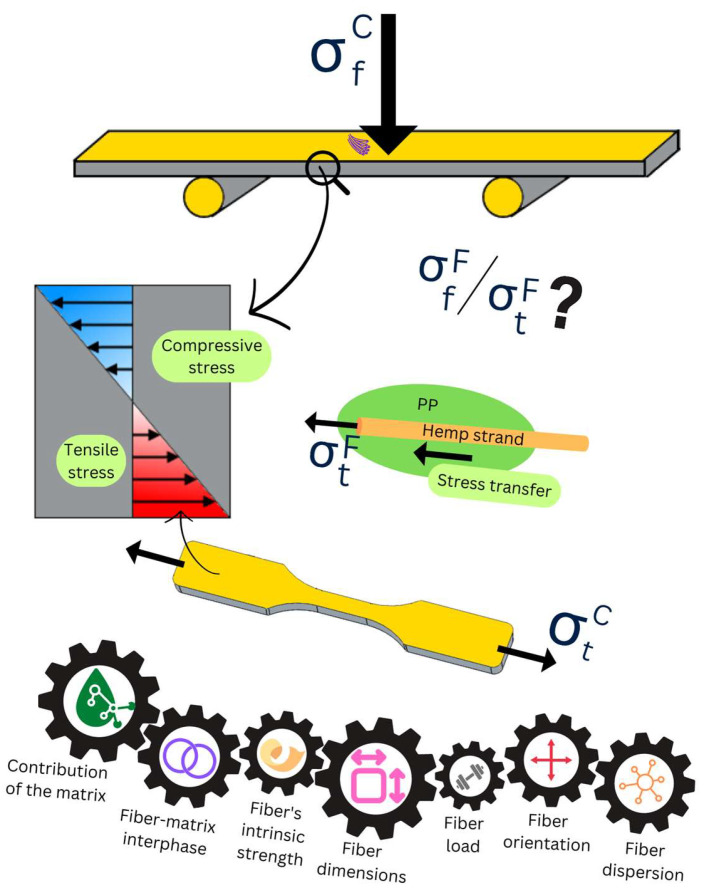
Schematic representation of bending tests (bottom), its relation to tensile tests (bottom), the matrix-to-fiber stress transfer, and factors affecting composite tensile/flexural strength.

In principle, the applications of natural fiber-reinforced PP encompass as many possibilities as PP/GF, i.e., construction materials, aerospace and automobile parts, and home appliances, among others [6,7]. The biomedical applications of PP-based composites, comprising ramie fiber and either hemp or coir fiber, have also been explored [8]. Nonetheless, natural fibers are relegated to a third position in the fiber-reinforced composites market, behind not only GF but also carbon fiber, partly due to their different intrinsic strength [9]. For instance, biocomposites usually possess lower tensile strength, as is well-known. Their flexural strength often receives less attention, even though the behavior against bending loads is more important in many cases of material design, such as flooring and roofing, than their capability to withstand outward forces only [10]. In fact, the flexural stress that a material undergoes can be expressed as a sum of compressive and tensile stresses, depending on the position along the axis of the force applied (Figure 1). In any case, both the tensile strength and the flexural strength of a thermoplastic material can increase by the effect of reinforcement fibers, due to the transfer of stress from the matrix to the fiber. Figure 1 also provides a simplified depiction of this phenomenon, described in detail elsewhere [11], and it displays the factors that determine the success or the extent of the improvement.

When it comes to the (comparatively) poor performance of plant fiber-reinforced composites when subjected to flexural stresses, a frequently alleged explanation is the lack of cohesion between the lignocellulosic fibers and the thermoplastic matrix [9,12]. Indeed, the macromolecules constituting plant fibers, mainly cellulose, are profusely hydrogen-bonded among themselves and with structural water [13]. In contrast, hydrophobic plastics such as polypropylene (PP) can neither accept nor donate hydrogen bonds, and the surface of PP is essentially non-polar with low electron donor capacity (1.9 mJ/m^2^) [14]. To overcome this difference, one of the most successful compatibilizers is maleic anhydride-grafted polypropylene (MAPP). Its maleic anhydride units easily form ester bonds with the hydroxyl groups of cellulose and/or hemicellulose, while its PP backbone offers proper compatibility with unmodified PP [15].

Due to the ease of management and harvest of hemp, some have argued that the relaxation of restrictions on their growth in the U.S. led to the so-called “hemp boom” in 2019, with many crops being wasted due to insufficient demand [16]. Since this demand is expected to increase for the purpose of fiber-reinforced composites, researchers must play their part to elucidate the most suitable manufacturing conditions.

Composites comprising PP and strands or bast fibers of hemp have already been reported with diverse impacts on mechanical properties [17,18]. For an overview of the most relevant advances in natural fiber-reinforced PP, the reader is referred to recent review articles [19,20]. Many different methods of composite preparation have been reported, such as lay-up, compression molding, injection molding and resin transfer molding. The present work opted for injection molding, due to its capability to accept high fiber loads [19] and its low cost [21].

Despite the extensive literature on PP/natural fiber composites, regarding composite characterization, most works are focused on the tensile properties and/or impact strength. A thoughtful analysis of the flexural strength behavior, assessing its dependence on the proportions of reinforcement fibers and compatibilizer, is still pending. This work addresses this knowledge gap, resorting to the modified rule of mixtures to evaluate the intrinsic flexural strength of hemp strands. Likewise, we discuss its relationship with its intrinsic tensile strength and reinforcement efficiency in comparison with sized GF.

## 2. Materials and Methods

### 2.1. Materials

As the matrix of all composites reported in this work, we used PP from Repsol (Repsol Polímeros SA, Sines, Portugal), product ISPLEN^®^ PP 090 G2M. Its density is 905 kg/m^3^ and its melt flow index is 35 g/10 min (230 °C, 2.16 kg). The MAPP coupling agent was Eastman G-3015 (Eastman Chemical Barcelona SL, Spain), with acid number 15 and density 913 kg/m^3^, M_w_ = 47,000 g/mol, and M_n_ = 24,800 g/mol. Hemp (*C. sativa L.*) bast fiber strands (HSs) were supplied by Agrofibra S.L. (Puigreig, Spain). For the sake of comparison with natural fibers, we used sized E-fibreglass Vetrotex^®^ (Saint-Gobain Weber Cemarksa SA, Montcada i Reixac, Spain).

The reagents used in the characterization of HSs were acquired from Scharlab SL (Sentmenat, Spain). For the titrations for surface polarity, we used methylglycol chitosan (MGCh) as the cationic polyelectrolyte and poly(vinyl sulfate) as the anionic polyelectrolyte, both from Wako Chemicals, GmbH (Neuss, Germany).

### 2.2. Experimental Methodology

#### 2.2.1. Analysis and Pretreatments of Constituents

Using a paper-cutter, HSs were fractionated to lengths of 10.0 ± 0.5 mm. They were suspended in cold water under slow overhead stirring (50–100 rpm) and the remaining hemp core was removed by flotation. An additional washing cycle took place for 20 min at 400–600 rpm. After filtering, washed strands were dried for 24 h at 80 °C. Then, HSs had their composition assessed according to TAPPI standards [22] typically employed for the chemical characterization of wood or lignocellulosics. This encompassed ashes (T 211 om-22), solvent extractives (T 204 cm-17), hemicellulose (T 249 cm-21), cellulose (T 429 cm-10), acid-insoluble or Klason lignin (T 222 om-15), and acid-soluble lignin (UM 250).

For purposes of comparison, a fraction of the strands was placed in a jacketed reactor with temperature control, suspended in distilled water at a consistency of 2 wt.%, and boiled for 60 min at 100 °C under atmospheric pressure. We estimated the surface polarity of untreated hemp strands (UHSs), boiled hemp strands (BHSs), hemp core, sized GF, and PP by performing a colloidal back titration [23]. For that, a known mass of strands or pellets was suspended in water, excess MGCh was added, vigorous mixing took place for 45–60 s with a stainless-steel overhead stirrer, and the heterogeneous mixture was separated by centrifugation (2000× *g*, 15 min). The free solution (supernatant) was titrated with the anionic polyelectrolyte, using toluidine blue O as an indicator.

#### 2.2.2. Production and Characterization of Composites

PP pellets were combined with 20, 30, 40 and 50 wt.% dry UHSs in a heated roll mixer (Iqap Masterbatch Group SL, Ibi, Spain), at 180 ± 5 °C and for 10 min. Increasing the load of the reinforcement phase beyond 50 wt.% severely decreased the melt flow index, hampering processability. The machine included two parallel rolls set at different angular speeds, 23 rpm and 29 rpm. MAPP was directly added in amounts of 0, 2 g, 4 g, 6 g, and 8 g per 100 g of dry fiber. Then, the blend was homogenized and granulated by grinding in a knife mill.

Composite pellets were kept at 80 °C and after 24 h, specimens for mechanical tests were prepared by injection molding. A Meteor 40 injection machine (Mateu & Sole SA, Barcelona, Spain) was employed, following the ASTM standard D3641 [24]. The rate was 45 cm^3^/s with the screw rotating at 300 rpm. The temperature in each of the three heating areas was set at 180 °C, 180 °C, and 200 °C. The pressure was 7.5 MPa during the volumetric phase and 3 MPa during the pressure maintenance phase. At least five strip shape specimens (127 mm × 12.7 mm × 3.2 mm) were produced for bending tests [25].

Prior to testing, specimens were conditioned at 23 °C and 50% relative humidity, as indicated by ASTM D618 [26]. We performed three-point flexural tests for the strip-shape samples by means of a Universal Testing Machine, model 1122, from Instron (Barcelona, Spain). This instrument was equipped with a 5 kN load cell and the strain rate was 0.10 mm/mm/min, according to the ASTM standard D790 [27]. The two supports were located 50 mm from each other.

After bending tests, specimens were subjected to scanning electron microscopy (SEM), using a ZEISS DSM 960A instrument (ZEISS Iberia, Madrid, Spain), carbon coating, a secondary electron detector, and a voltage of 7 kV. For comparison purposes, micrographs were obtained from the fracture section of both PP/HS composites without compatibilizer and samples with the optimal proportion of MAPP.

**Figure 2:**
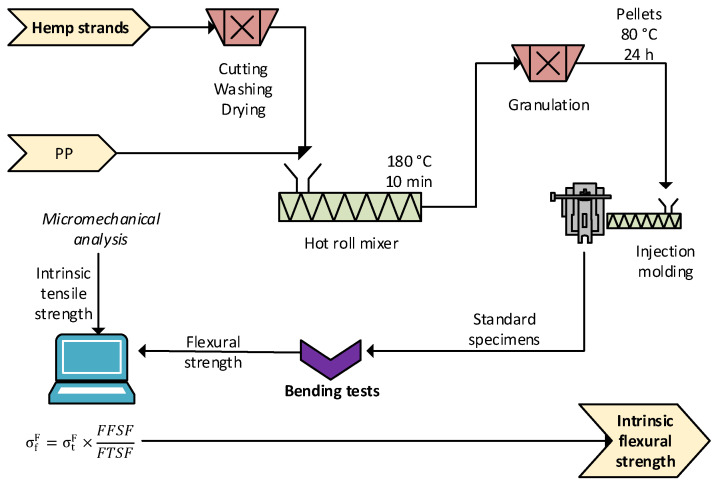
Diagram of the methodology, including blending, injection, and bending tests (to be combined with outputs from the tensile tests [28]).

A diagram of the experimental procedure is displayed in Figure 2.

### 2.3. Calculation Methodology

It is customary to split the contributions of the reinforcement fibers and matrix to the tensile strength of the composite (σ_t_^C^) in terms of the modified rule of mixtures [29,30]:

σ_t_^c^ = f_c_ × σ_t_^F^ × V^F^ + (1 − V^F^) × σ_t_^m^ (1)

where V^F^ is the volume fraction of fibers, σ_t_^F^ is the strength of the matrix at composite failure, σ_t_^F^ is their intrinsic tensile strength, and f_c_ is known as the coupling factor. The product of the latter two parameters, f_c_ and σ_t_^F^, is referred to as the fiber tensile strength factor (FTSF) [31].

An analogous equation has been reported in other works for the analysis of the composite’s flexural strength (σ_f_^C^) at the level of its constituents [32]:

σ_f_^C^ = f_c_^f^ × σ_f_^F^ × V^F^ + (1 − V^F^) × σ_f_^m^* (2)

where f_c_^f^ is the efficiency factor under flexural stress and σ_t_^m^* is the flexural strength of the matrix at the maximum strain attained by the composite. f_c_ and f_c_^f^ depend on the quality of the interphase, the orientation of the fibers, and their dimensions. In a similar way to Equation (1), the product f_c_^f^ × σ_f_^F^ is the fiber flexural strength factor (FFSF). Its value can be estimated from the slope of (1 − V^F^) × σ_f_^m^* against V^F^. Then, with the approximation f_c_ ~ f_c_^f^, it can be seen that the ratio between the intrinsic flexural and tensile strengths of fibers equals the ratio between the aforementioned factors:

(3) $$\sigma_{f}^{F}=\sigma_{t}^{F}\times \frac{\mathrm{FFSF}}{\mathrm{FTSF}}$$

In another context, the reinforcement efficiency parameter [33] for flexural strength, η_σ_^f^, can be derived from rewriting Equation (2) as:

σ_f_^c^ = σ_f_^m^ + (η_σ_^f^ σ_f_^F^ − σ_f_^m^) V^F^ (4)

In plain terms, between two kinds of reinforcement fibers that attain the same enhancement in flexural strength, the one of lower intrinsic strength is the most efficient.

## 3. Results and Discussion

### 3.1. The Relevance of the Composition and Polarity of Hemp Strands

Even in the absence of chemical treatments, UHSs possess high proportions of cellulose, as shown in Table 1. α-Cellulose and hemicellulose contents account for a total holocellulose percentage as high as 85.7%. This explains why their surface was much more polar than that of PP (Table 2). During surface polarity assays, MGCh was able to become adsorbed on their hydroxyl groups by ion-dipole interactions, while PP and sized GF were only capable of establishing dispersive interactions. Likewise, significant dipole-dipole interactions cannot be expected between PP, whose effective dipole moment is roughly 0.05 D [34], and the surface of HSs, much more prone to self-bonding. In general, this self-bonding, which may lead to agglomeration and poor dispersion, is the main difficulty that researchers face when using cellulosic reinforcements for plastics [35].

Although in a lesser proportion than cellulose, one of the greatest contributors to the polarity of fiber surfaces is pectin, whose galacturonic acid units are quantitatively deprotonated in neutral aqueous media [36]. Pectin, along with other gums, is included among hot water extractives (3.2 wt.%, Table 1). Consequently, a boiling treatment significantly reduced the polarity of HSs (24.08 µeq MGCh/g, Table 2). Regarding the hemp core, while it has more lignin than the bast, it also possesses more gums and hemicellulose [37].

**Figure 3:**
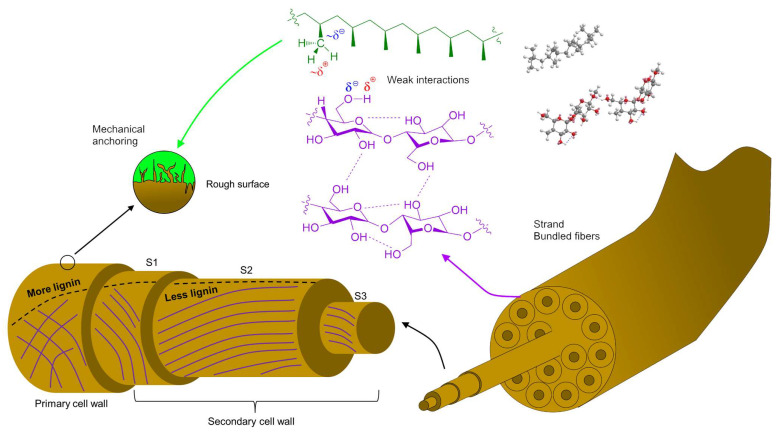
Structure of hemp strands, their constituting fibers, the qualitative profile of lignin content (dashed line), and their plausible interactions with the thermoplastic matrix.

Nonetheless, besides these considerations on individual macromolecules and their functional groups, surface polarity is highly influenced by their interactions, their distribution across the fiber, and the morphology and surface area of fibers. For instance, their lignin content (totaling 5.1 wt.%) is relatively small, at least when compared to hemp core or to any kind of wood fibers [38], but it is known to be more abundant in the outer layers of the fibers (middle lamellae, primary walls) than across the secondary walls (Figure 3). Furthermore, cellulosic fibrils in the primary wall display diverse orientations, giving way to pores and to certain surface roughness that is required for the mechanical anchoring of the fiber into the matrix. In contrast, fibrils are arranged at 60–80° angles in the first layer of the secondary wall (S1 layer), and almost parallelly to the axis in the S2 layer [39].

Lignin, like the polyphenolic compounds that are included in the fraction of ethanol-benzene extractives (Table 1), is less polar than carbohydrates and more polar than the thermoplastic matrix. Hence, on one hand, they help regulate the polarity of the surface of fibers (Table 2) [40]. On the other hand, lignin, hemicellulose, and carbohydrates are mostly amorphous components, affecting the intrinsic strength of reinforcement fibers [6].

Considering the relevance of composition, polarity, and available surface area, Figure 3 depicts the main components of HSs and schematizes fiber-matrix interactions. Given the great difference in polarity, it is expected that the reinforcement fibers are preferably self-bonded instead of bonded to PP. Without compatibilizers, the fibers are mechanically anchored, but their intermolecular interactions with the matrix are limited to weak dispersive forces. In this sense, the rough and porous surface of the strands plays an essential role in stress transfer. It is known that in composites encompassing both weak interfacial interactions and smooth fiber surfaces, the formation of voids and cracks transversely to the stress direction is eased, thus attaining strength values below that of the non-reinforced matrix [41].

### 3.2. Evaluation of the Dose of Compatibilizer

**Figure 4:**
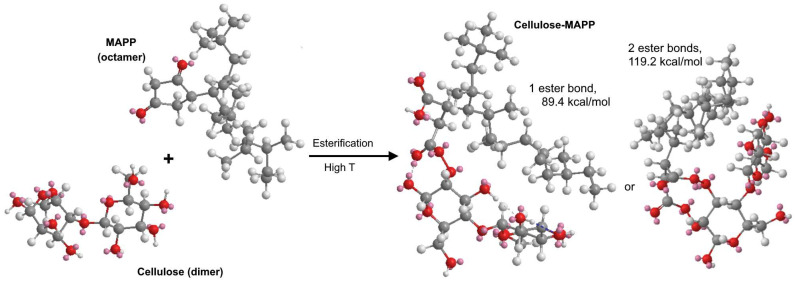
Computational optimization (Chem3D Pro’s *MM2* energy minimization calculations) of the conformation of MAPP after esterification with cellobiose, chosen as a proxy molecule for the reinforcement fibers. Grey: C. White: H. Red: O.

Ignoring steric effects and torsional strain limitations, the stoichiometric ratio of hydroxyl groups to maleic anhydride units is 2, i.e., each anhydride unit can form two ester bonds. Alternatively, if only one is generated, the resulting carboxyl group at the other end of the maleic moiety is available for hydrogen bonding with a neighbor hydroxyl group. We tested the two possibilities in *Chem3D Pro*, using cellobiose as a proxy for cellulose or hemicellulose. MAPP was modeled as isotactic PP with eight repeating units and one grafted maleic anhydride unit. Then, molecular mechanics calculations were run aiming at energy minimization. The total free energy was 89.4 kcal mol for the case of one ester bond and 119.2 kcal/mol for two ester bonds, partially due to the high torsion energy in the latter case (28.9 kcal/mol). Figure 4 shows the resulting optimized structures.

It is important to note that only a small fraction of the hydroxyl groups in HSs are available for esterification with MAPP. Most of them are H-bonded in intramolecular or intermolecular interactions, or physically not accessible due to being located beneath the surface of the fibers.

Those –OH groups on the surface can be roughly estimated under the following approximations and inputs: (i) the surface consists entirely of cellulose I with similar proportions of cellulose Iβ (monoclinic) and cellulose Iα (triclinic) [42,43]; (ii) the specific surface area (*SSA*) is 0.8 m^2^/g; (iii) the rotation around glycosidic bonds is neglected; (iv) the (200) plane [44] is consistently parallel to the surface; (v) considering the unit cell of anhydroglucose dimers [43], the area of the (200) planes in each unit cell (*S*_200_) is 6.94 × 10^−17^ m^2^. The number of superficial hydroxyl groups per gram (*N_OH_^sup^*) is then:

(5) $$N_{\mathrm{OH}}^{\sup}\left(-{\mathrm{OH}\;g}^{-1}\right)=\frac{\mathrm{SSA}\left(\mathrm{fiber}\right)}{S_{200}\left(\mathrm{dimer}\right)}\times \frac{6-\mathrm{OH}\left(\mathrm{dimer}\right)}{\mathrm{Molecular}\;\mathrm{weight}}\times N_{A}$$

where *N_A_* is the Avogadro constant. Under these assumptions, according to Equation (5), there are roughly 5.6 × 10^21^ accessible hydroxyl groups per gram of fiber. It can be noted that if the factor *SSA*/*S*_200_ is removed from Equation (5), then the result is the total number of hydroxyl groups across the cellulosic material. Hence, this ratio equals that of superficial –OH groups to the total number of –OH groups, resulting in 0.062%.

**Figure 5:**
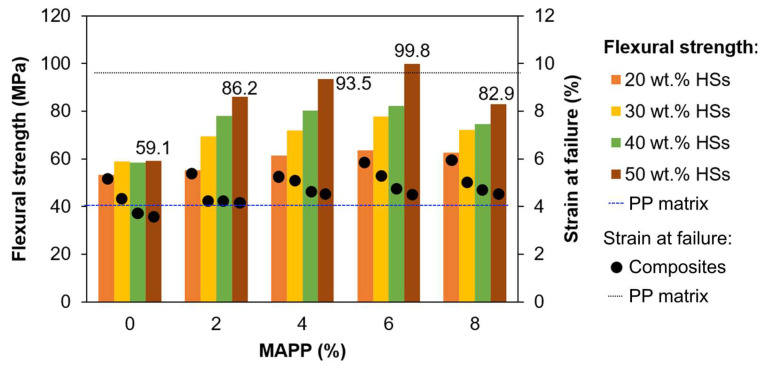
Evolution of the flexural strength (columns) and the strain at composite failure (dots) for PP/HS composites, as functions of the ratio of compatibilizer to fiber. Number labels correspond to the highest fiber load. Dashed lines show the flexural strength and maximum strain of the matrix.

It is easy to see that this estimation is subjected to diverse sources of error, e.g., assuming that the surface of the fibers is solely constituted by crystalline cellulose. The optimum percentage of MAPP, in any case, must be determined experimentally. For that, Figure 5 shows the different values of flexural strength obtained for MAPP proportions of 0 to 8 g per 100 g of strands. Even without MAPP, there was a significant increase in flexural strength over that of the matrix (40.2 MPa), due to the fibers’ rough surfaces attaining proper fiber-in-matrix anchoring. Likewise, with maximum strain percentages below 6%, the composite became more brittle in comparison to the matrix (9.6%).

Data for the highest fiber load, 50 wt.% hemp, are labeled in Figure 5 for the case of exemplification, but the trends with the addition of MAPP are qualitatively identical for all levels of reinforcement. In short, differences in the strain at composite failure are hardly significant, but flexural strength was notably affected. As expected, the higher the fiber load, the greater the improvement attained by MAPP, since more stress can be transferred to fibers that, without compatibilizer, did not possess that capability; 2% MAPP (i.e., 2 g per 100 g of HSs) attained a 46% improvement from PP/HSs (50 wt.%). Flexural strength increased less abruptly towards 6 g of MAPP per 100 g of hemp, reaching a 69% improvement over PP/UHSs without MAPP, and then it decreased when the proportion of MAPP was 8%. This excessive amount of MAPP likely decreased the overall crystallinity of the matrix, thus being detrimental to the performance of PP/hemp composites in bending tests [45]. As recently reported by Yamaguchi et al. [46], the crystallinity of the compatibilizer is more important than the dosage when it comes to the interfacial shear strength.

It may be concluded that the optimum proportion of MAPP is 6%, i.e., 0.06 g per gram of fiber. In this case, one-way ANOVA tests confirmed the significance of the difference over the composites without MAPP (*p* = 0.022).

**Figure 6:**
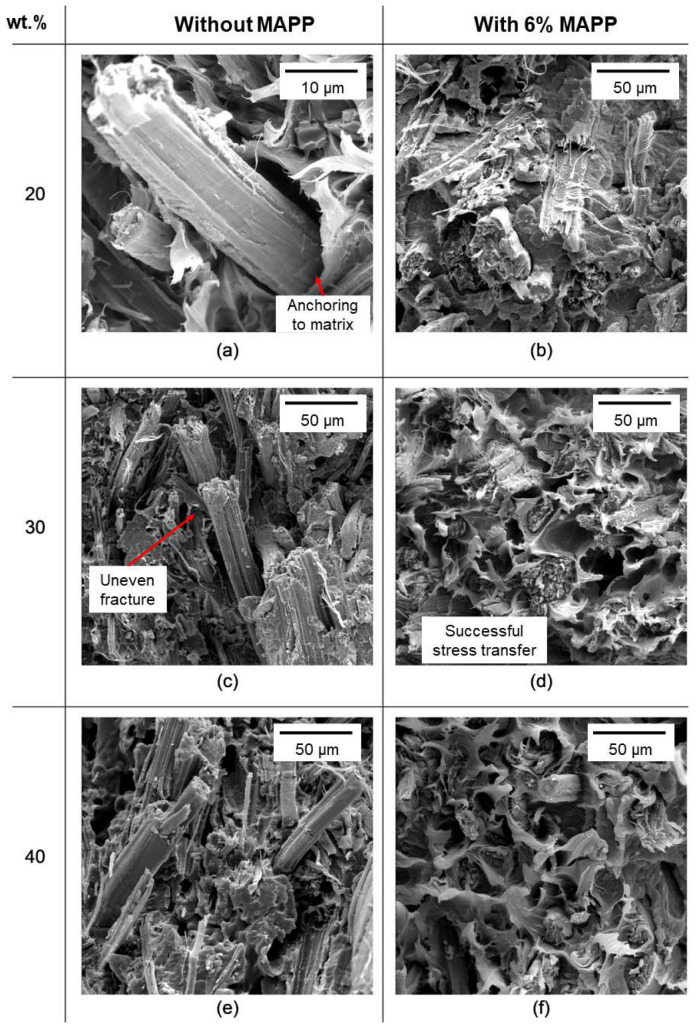
Cross-sectional SEM images of PP/hemp composite specimens after fracture, encompassing different fiber loads and whether or not MAPP was used as compatibilizer; (**a**) 20wt% composite without MAPP, (**b**) 20wt% composite with MAPP, (**c**) 30wt% composite without MAPP, (**d**) 30wt% composite with MAPP, (**e**) 40wt% composite without MAPP, (**f**) 40wt% composite with MAPP.

The micrographs in Figure 6 evidence the improvement imparted by the incorporation of MAPP. In Figure 6a a truncated fiber is shown at a high level of magnification to highlight its mechanical anchoring into the matrix. A composite with the same fiber load but with MAPP displays a higher degree of integration with the matrix in Figure 6b. Furthermore, when comparing Figure 6c,e (no MAPP) with Figure 6d,f (6% MAPP), respectively, the former display uneven fracture surfaces, as the failure of the matrix took place without transferring the stress along the whole length of the fibers. In contrast, the presence of MAPP propitiated both the dispersion of fibers and their capability to accept tensile and compression loads.

### 3.3. Enhancement of Flexural Strength and Potential to Replace GF

By reinforcing PP with HSs with the highest fiber load (50 wt.%) and the optimum amount of MAPP (6 g per 100 g of fibers), the flexural strength was more than doubled. Specifically, σ_f_^C^ was enhanced by up to 148% (Table 3). As a limitation, the strain at failure (ε_f_^C^) decreased by the effect of the reinforcement. In other words, the composite became increasingly brittle. This is a long-known, nearly ubiquitous effect of discontinuously reinforced composites [47]. When evaluated against the volume fraction of reinforcement fibers, it can be seen that σ_f_^C^ values follow a linear trend (Pearson’s r > 0.95). This feature, such as in the case of the tensile strength, indicates good quality of the matrix-fiber interphase and compliance with the modified rule of mixtures [48].

The mean intrinsic tensile strength of fibers (σ_t_^F^), as estimated in a previous work of ours from micromechanical models [28], is 556 ± 68 MPa, within the range of values widely reported in the literature (368–800 MPa) [49]. In a first, preliminary approximation, the σ_f_^F^/σ_t_^F^ ratio can be estimated as the ratio of the values corresponding to the composites, i.e., σ_f_^C^/σ_t_^C^. This way, taking into account all levels of fiber load, the tensile/flexural ratio ranges from 1.46 (0%) to 1.83 (20 wt.%). The subsequent mean intrinsic flexural strength is then comprised between 711 MPa and 1144 MPa.

It is more accurate though to estimate σ_t_^F^ as in Equation (3), after calculating both FTSF (126) and FFSF (216). The ratio FFSF/FTSF is 1.71, which is higher than that of softwood fibers, 1.48 [32]. Unlike softwood fibers, HSs have high cellulose content and a low percentage of amorphous components without the need for aggressive chemical treatments. In any case, the mean intrinsic flexural strength of HSs with 6% MAPP is 953 ± 116 MPa. Using this value in Equation (2) to obtain the coupling factor, f_c_^f^ is calculated as 0.227. According to Sanadi et al. [50], a factor of 0.2 or close to 0.2 evidences a properly bonded system.

In comparison, as expected, PP/sized GF composites attained significantly higher enhancements over the matrix than PP/HS. Table 4 shows the improvements of sized GF up to 40 wt.%. At this level of fiber load, PP reinforced with hemp strands but without MAPP (PP/UHS), along with PP/HS with the optimum amount of compatibilizer, are also displayed. It can be remarked that with 40 wt.% reinforcement fibers, the σ_f_^C^ of PP/UHS, that of PP/MAPP (6%)/HS and that of PP/sized GF surpassed the σ_f_^C^ of the matrix by 45.5%, 104% and 161%, respectively.

For sized GF, *FTSF* is 287 and *FFSF* is 472. Its intrinsic tensile strength is *σ_t_^F^* = 2415 MPa [51]. From the *FFSF*/*FTSF* ratio, 1.64, we can estimate its intrinsic flexural strength as *σ_f_^F^* = 3961 MPa, which is very close to the values reported elsewhere [33]. However, it should be noted that, while the enhancements attained by sized GF were greater, the difference in *σ_f_^C^* is not proportional to the difference in *σ_f_^F^*. In fact, addressing the efficiency of HSs as reinforcement [33], a linear fitting to Equation (4) yields an average value of η_σ_^f^ = 0.209. For GF, the efficiency parameter was found to be lower, η_σ_^f^ = 0.045.

## 4. Conclusions

This work sought composites consisting of a conventional thermoplastic matrix (PP) and natural fibers (hemp strands) to assess their performance under flexural stress in comparison to GF. The optimum of compatibilizer, 0.06 g of MAPP per gram of fibers, was determined experimentally, as it corresponded to the dose that consistently yielded the highest increase in flexural strength. For the same fiber load (40 wt.%), the composite PP/HS with the optimal amount of compatibilizer attained a flexural strength value as high as 82.2 MPa, close to that of the conventional material PP/sized GF (105 MPa). While keeping the optimal proportion of MAPP, improvements as high as 148% could be reached by increasing the load on hemp strands to 50 wt.%.

Overall, the best possible combinations comprised 40–50 wt.% of hemp strands, 2–3 wt.% of MAPP, and 47–58 wt.% of PP. Nonetheless, the strain at failure of the resulting materials decreased from 9.6% to 4.6–4.8%. Despite the promising results of PP/HS, the flexural strength of PP/GF materials was greater, due to the high intrinsic flexural strength of GF (3961 MPa). However, hemp strands were found to be a more efficient reinforcement for PP (η_σ_^f^ = 0.209) than the most conventional reinforcement fiber, GF (η_σ_^f^ = 0.045).

## Figures and Tables

**Table 1:** Chemical composition of hemp strands as measured from TAPPI methods. Tolerance intervals encompass twice the standard deviation.

**Ash (wt.%)**		2.7 ± 0.7
**Extractives (wt.%)**	Hot water	3.2 ± 0.2
	Ethanol-benzene (1:2)	5.1 ± 0.5
**Lignin (wt.%)**	Acid-soluble	3.9 ± 0.3
	Acid-insoluble (Klason)	1.22 ± 0.08
**Holocellulose (wt.%)**	Cellulose (α-cellulose)	74.2 ± 2.3
	Hemicellulose	11.3 ± 1.2

**Table 2:** Surface polarity of constituent materials, estimated from colloidal titrations. Results with boiled strands and hemp core are displayed for comparison purposes.

**Matrix polarity (µeq MGCh/g)**	PP	4.56
**Fiber polarity (µeq MGCh/g)**	UHSs	29.70
	BHSs	24.08
	Hemp core	34.10
	Sized GF	4.46

**Table 3:** Strain at composite failure and flexural strength of PP/hemp composites, highlighting the percentage enhancements over the PP matrix. Tolerance intervals encompass twice the standard deviation.

Material	Reinforcement	V^F^	σ_f_^C^ (MPa)	Δσ_f_^C^ (%)	ε_f_^C^ (%)
PP	0 wt.%	0	40.2 ± 1.0	--	9.6 ± 0.2
PP/HSs with 6% MAPP	20 wt.%	0.132	63.6 ± 0.9	58.2	6.0 ± 0.3
	30 wt.%	0.206	77.7 ± 0.9	93.3	5.3 ± 0.2
	40 wt.%	0.288	82.2 ± 1.3	104	4.8 ± 0.2
	50 wt.%	0.377	99.8 ± 1.1	148	4.6 ± 0.3

**Table 4:** Tensile strength, flexure strength, and maximum strain in both tests of PP/sized GF composites, in comparison to PP/HS, displaying the percentual enhancement over the matrix in each case. The amplitude of the tolerance intervals is twice the standard deviation.

Material	Reinforcement	V^F^	σ_t_^C^ (MPa)	Δσ_t_^C^ (%)	ε_t_^C^ (%)	σ_f_^C^ (MPa)	Δσ_f_^C^ (%)	ε_f_^C^ (%)
PP/sized GF	20 wt.%	0.084	50.9 ± 0.9	84.4	3.1 ± 0.1	78.0 ± 2.7	94.0	4.6 ± 0.2
	30 wt.%	0.136	58.5 ± 4.3	112	3.0 ± 0.2	88.1 ± 3.1	119	3.3 ± 0.1
	40 wt.%	0.197	67.1 ± 1.7	143	2.4 ± 0.1	105 ± 1.3	161	2.4 ± 0.1
PP/UHS	40 wt.%	0.288	32.8 ± 0.9	18.8	3.5 ± 0.1	58.5 ± 0.4	45.5	3.7 ± 0.2
PP/HS with 6% MAPP	40 wt.%	0.288	48.7 ± 1.1	76.4	3.5 ± 0.3	82.2 ± 1.3	104	4.8 ± 0.2

## Data Availability

All data explicit in the manuscript or else available at request.
